# Ensemble-Based Model-Agnostic Meta-Learning with Operational Grouping for Intelligent Sensory Systems

**DOI:** 10.3390/s25061745

**Published:** 2025-03-12

**Authors:** Mainak Mallick, Young-Dae Shim, Hong-In Won, Seung-Kyum Choi

**Affiliations:** 1 G. W. Woodruff School of Mechanical Engineering, Georgia Institute of Technology, Atlanta, GA 30332, USA; mmallick7@gatech.edu (M.M.); young-dae.shim@me.gatech.edu (Y.-D.S.); 2 Department Smart Manufacturing Technology, Sungkyunkwan University, Suwon-si 16419, Gyeonggi-do, Republic of Korea; 3 Korea Institute of Industrial Technology, Cheonan-si 31056, Chungcheongnam-do, Republic of Korea

**Keywords:** few-shot learning, model-agnostic meta-learning (MAML), transformer, autoencoder, ensemble learning, digital twin, industrial robot (IR), predictive maintenance (PdM)

## Abstract

Model-agnostic meta-learning (MAML), coupled with digital twins, is transformative for predictive maintenance (PdM), especially in robotic arms in assembly lines, where rapid and accurate fault classification of arms is essential. Despite gaining significant traction, the framework faces significant challenges, like hypersensitivity to learning parameters and limited generalization during meta-testing. To address these challenges, we proposed an ensemble-based meta-learning approach integrating majority voting with model-agnostic meta-learning (MAML), and operational grouping was implemented via Latin hypercube sampling (LHS) to enhance few-shot learning ability and generalization along with maintaining stable output. This approach demonstrates superior accuracy in classifying a significantly larger number of defective mechanical classes, particularly in cross-domain few-shot (CDFS) learning scenarios. The proposed methodology is validated using a synthetic vibration signal dataset of robotic arm faults generated via a digital twin. Comparative analysis with existing frameworks, including ANIL, Protonet, and Reptile, confirms that our approach achieves higher accuracy in the given scenario.

## 1. Introduction

Modern manufacturing plants are highly automated and robotized, which replaces human labor in repetitive and hazardous tasks involving heavy loads. In addition to human safety, the application of industrial robots has an impact on productivity, as robots can be operated 24/7 for an extended period of time. Even though recent mechanical components have reliable properties, material defects, operating conditions, or other environmental factors force the degradation of the robot. These effects lead to tool position deviation or the vibration of the robotic structure, which reduces the quality of the manufactured products. In order to prevent the manufacturing of defective products, a prediction of the maintenance period of robots is required.

General maintenance operations are usually conducted after a failure has occurred or in a fixed time interval. However, this could reduce the company’s profits due to unexpected production line stoppages or the premature replacement of undamaged parts. With the development of sensor technology, sensors have been integrated into mechanical products to monitor and gather data, such as temperature, humidity, acceleration, voltage, etc., which can be used for predicting the condition state [1]. Predictive maintenance (PdM) for industrial robotics is a promising approach for predicting remaining-useful-life (RUL) or failure events to flexibly schedule the maintenance period. It has emerged as a critical application in Industry 4.0, leveraging AI to anticipate and schedule equipment maintenance, thereby improving operational efficiency and reducing downtime [2]. The integration of smart sensors and digital twins within the Metaverse further enhances predictive maintenance by enabling the continuous monitoring of real-world assets in virtual environments, allowing for early fault detection and intervention [3]. Additionally, wearable inertial sensors play a crucial role in predictive maintenance for robotic systems by tracking fine motor movements and identifying deviations that may indicate potential failures. These advancements collectively contribute to a more proactive and intelligent approach to maintenance, reducing costs and increasing system reliability across various industries [4].

Due to the complexity of mechanical systems, such as industrial robots, data-driven approaches are becoming popular for their excellence in fault detection and their ability to facilitate access to large amounts of data due to sensor technology [5]. The health condition of a spot-welding robot has been predicted by using the torque data of each joint of the robot [6]. Support vector machine (SVM) and extreme learning machine (ELM) algorithms were used, which have excellent prediction results for nonlinear systems [7]. The current signals of the robot system can be used to predict the degradation of the motion accuracy [8]. Segmented time series current signals were labeled with robot arm accuracy and are used for the training data. The featured data showed a good correlation with trajectory accuracy. In addition to the current signal, vibration can be a valuable source for detecting degradation of performance for industrial robots [9]. A one-dimensional convolutional neural network (1D-CNN) has been used to classify health states, such as normal operation, collision, floor surface condition, or operational speeds. The 1D-CNN shows a high capability to utilize the time series sensor signals.

However, traditional predictive maintenance systems rely heavily on extensive labeled datasets, which are often unavailable in industrial environments. This limitation hampers effective fault diagnosis and proactive maintenance, particularly in scenarios where the data are scarce or imbalanced. Another issue of processing this type of data is the effective capture of the finer complexities and patterns when they are converted into images; some methods, like multi-sensor fusion with PCA, were developed to mitigate this to some extent [10]. After converting the signal to images, meta-learning approaches, such as model-agnostic meta-learning (MAML), have been used to address these challenges by enabling models to generalize across tasks and adapt quickly to limited data [11,12]. A significant application of these methods is in the predictive maintenance of robotic arms, where the accurate classification of mechanical faults is essential to minimize downtime and ensure system reliability [13].

Despite its potential, two primary challenges hinder the effective application of MAML in industrial robotics. First, the hypersensitivity of the model to its learning parameters poses a significant bottleneck. Parameters, such as the inner-loop learning rate, outer-loop learning rate, batch size, and the number of adaptation steps, greatly influence the stability of MAML [14]. During meta-training, the model’s performance often oscillates or diverges as the number of epochs increases, primarily due to the intricate interplay between these hyperparameters. This instability makes it difficult to achieve consistent generalization across tasks, particularly in environments characterized by noisy and imbalanced data.

Second, the limited information intake during the meta-test phase further complicates the effective deployment of MAML in industrial applications. In industrial-grade robotic systems, mechanical faults are rare events that occur after prolonged degradation processes, resulting in monitoring data predominantly skewed toward healthy system conditions. During meta-testing, the support set typically contains very few examples of fault classes, limiting the model’s ability to capture sufficient distinguishing features. For this anomaly, the imbalance ratio (IR), defined as the ratio between a number of samples for the majority class to the minority class, becomes relatively high (10–20) compared to the normal dataset. This results in further suboptimal generalization on the query set and negatively impacts the classification accuracy for unseen fault scenarios.

Efforts to address these challenges have included data augmentation techniques, such as time-series transformations and generative adversarial networks (GANs) [15], and various few-shot learning frameworks, like ProtoNet and relational networks [16]. Optimization-based approaches, such as MAML and its variations (ANIL, Reptile), have also been explored extensively [17,18]. While these methods have shown promise, especially because of few-shot learning’s explicit ability to normalize class imbalance with its limited fault class sample intake (1~10 samples in most cases) in support sets [19,20], they are often constrained by either the instability of the training process or the limited utility of the support set during the meta-test phase [21]. This is particularly problematic in cross-domain few-shot learning scenarios, where the model must adapt to highly diverse fault conditions with minimal labeled data.

To bridge these gaps, we propose an ensemble-based meta-learning framework that integrates MAML with an operational grouping strategy, i.e., ensemble MAML with an operational grouping (EMOG) method to enhance generalization and stability. The proposed approach mitigates the instability associated with individual models and improves fault classification accuracy by leveraging a majority voting mechanism across multiple models. Additionally, the operational grouping method ensures maximal information intake during the support set phase of meta-testing, addressing the issue of sparse and imbalanced fault data. These enhancements are particularly effective in industrial robotics, where multiclass sensor signal classification is critical for maintaining operational efficiency and minimizing downtime. The proposed framework is validated using synthetic signal datasets generated from a digital twin of a robotic arm in Isaac SIM. The synthetic sensor data were collected by the inertial measurement unit (IMU) sensor implemented in the digital twin. A convolutional autoencoder (CAE) was used to encode the time series or signal data into a latent representation and decode it into a structured image format. An autoencoder was used for this purpose because of its capacity to capture complex, non-linear structures in the sensor signal during image conversion, making it more suitable for this task when compared to more conventional techniques, like PCA [22,23]. These images were used for the meta-testing phase for the few-shot learning case.

The main contributions of our work are outlined as follows:

1. An ensemble-based model, the agnostic meta-learning method (MAML), is proposed using majority voting and operational grouping to maximize information intake in a few-shot scenarios.
2. A convolutional autoencoder-based multi-sensor to fused RGB image conversion method is implemented for converting senor signals to RGB images, and the images are later used for classifying different fault classes.
3. To the best of our knowledge, this work is the first of its kind to implement ensemble-based MAML algorithms with such diverse classes in a synthetic dataset.

The remainder of the paper is structured as follows. Section 2 provides details on the previous and related works, and Section 3 provides information on the framework and methodology. Section 4 provides a brief on the experimental setup, and Section 5 provides the results and comparisons with similar approaches. Finally, Section 6 provides the conclusions of this study.

## 2. Related Work

Predictive maintenance [24] in industrial robotics has evolved with the advent of advanced machine learning and deep learning techniques [25]. Generative adversarial networks (GANs) [26] have been explored to address the scarcity of fault data by generating synthetic datasets that mimic failure scenarios with digital twin models, a replica of the electromechanical system that can show the behavior and performance of that system in digital space, using real-time sensor data and simulations [27]. These synthetic datasets allow models to learn from a broader range of conditions, improving their ability to generalize. However, GANs often face challenges, such as mode collapse and the generation of low-quality synthetic samples, which can hinder model reliability when deployed in real-world scenarios [28].

Graph neural networks (GNNs) offer a novel approach by leveraging the topological structure of robotic systems. These networks model the interdependencies between various robotic components, such as joints and actuators, enabling more accurate fault diagnosis and failure prediction. Despite their promise, GNNs require significant computational resources and high-quality graph representations of the system, which can be challenging to obtain in industrial environments with legacy systems.

Long short-term memory (LSTM) networks, a type of recurrent neural network, are widely applied for predictive maintenance in robotics due to their ability to model temporal dependencies in sensor data. LSTMs excel in capturing patterns over time, such as gradual wear or irregular vibrations. However, they are computationally intensive, particularly when processing high-dimensional or long-term sequential data, and may suffer from vanishing gradient issues when not properly optimized.

A data-driven Takagi–Sugeno (T-S) fuzzy modeling approach has been proposed for incipient fault detection and isolation (FDI) in these types of systems, leveraging the total measurable fault information residual (ToMFIR) for fault identification. This method effectively monitors slowly developing and intermittent faults, demonstrating its applicability in predictive maintenance. However, its reliance on accurate system modeling and predefined fault patterns limits its adaptability to complex, highly dynamic industrial environments [29].

Each of these methods contributes uniquely to predictive maintenance but comes with its own limitations. GAN-based approaches struggle with synthetic data quality [30], GNNs require accurate system modeling and computational power, and LSTMs face challenges with scalability and computational cost [31]. These shortcomings highlight the need for hybrid or ensemble approaches that combine the strengths of these techniques to build more robust and efficient predictive maintenance frameworks for industrial robotics [32].

Several meta-learning approaches have been developed to tackle these bottlenecks with few-shot learning and rapid adaptation across tasks, with each introducing unique optimization techniques, as hinted in the previous section. We have compared our results to all the meta-learning algorithms described below to evaluate the effectiveness of our approach.

ANIL is a simplified version of MAML where only the classifier is updated while the feature extractor remains frozen. As a result, it significantly reduces computational complexity by eliminating second-order gradient calculations [33]. However, this simplification comes at the cost of adaptability, as it struggles when tasks require fine-tuned feature representations. Similarly, Reptile is a first-order meta-learning algorithm that updates model parameters by averaging gradients across tasks. Unlike MAML, it does not require second-order derivatives, making it more computationally efficient [34]. Nevertheless, since it lacks explicit task-specific adaptation, it performs poorly in highly diverse task distributions where fine-grained adjustments are necessary. In contrast, ProtoNet takes a metric-based approach by classifying new samples based on their distance to class prototypes in an embedding space. This method is highly effective for few-shot learning, particularly when data points form compact and well-separated clusters [35]. However, its reliance on well-defined clusters makes it vulnerable to high intra-class variance, leading to performance degradation in complex datasets. On the other hand, MAML optimizes quick learning by explicitly learning an initialization that allows fast adaptation to new tasks with only a few gradient updates. While this enables strong generalization, the method is computationally expensive due to the requirement of second-order gradients. Additionally, its performance is susceptible to hyperparameters, which can affect both stability and convergence [36].

## 3. Methodology and Overview

This section outlines the methodology adopted in this study, which is divided into two subsections. The first subsection focuses on the dataset generation process and the operational grouping method, designed to maximize information intake during the meta-test phase. This ensures that the model can effectively generalize by leveraging the most critical features of the data.

The second subsection introduces the proposed ensemble-based MAML framework. This subsection explains how the ensemble mechanism is integrated with MAML to address the hyperparameter instability commonly observed in meta-learning models.

### 3.1. Dataset Generation and Operational Grouping Strategy

Dataset generation and preprocessing were significant advancements in the entire workflow; in the following sections, we will discuss how we have implemented our operational grouping method, starting from digital twin’s creation.

**Figure 1:**
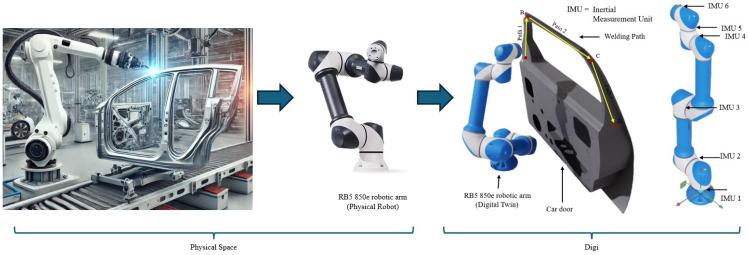
Digital twin creation workflow.

The digital twin of the RB5 850e robotic arm was developed using its unified robot description file (URDF) [37], which encodes structural and physical properties, such as joint limits, link dimensions, and inertial parameters. This URDF was imported into the NVIDIA Isaac Sim platform, which was selected for its high-fidelity physical simulation capabilities, including accurate modeling of forces, collisions, and dynamics. To ensure realistic behavior, a manual calibration process, known as the gain test, was conducted to fine-tune key physical parameters, such as force (*F*), friction coefficient (*µ*), stiffness (*k*), and damping (*b*). These parameters were essential for replicating the robotic arm’s motion and vibration dynamics, and were modeled using the following equation:

(1) $$\tau =k\theta +b\dot{\theta }+F_{f}$$

where *τ* is the joint torque, *θ* is the joint angle, $\dot{\theta }$ is the angular velocity, and *F_f_* = *µN* represents the frictional force, proportional to the normal force *N* and friction coefficient *µ*. This calibration ensured precise articulation and realistic joint behavior during the simulations [38]. Figure 1 provides an overview of the digital twin creation and the positions of the IMU sensors in the robot arm. Path A–D in the figure shows the path followed by a robot arm in typical welding action.

To collect detailed data, IMU sensors were strategically placed at critical joints of the robotic arm, including the base, shoulder, elbow, wrist1, wrist2, and TCP (tool center point). These sensors captured high-frequency motion and vibration data, including acceleration components (a_x_, a_y_, a_z_) and angular velocity components (ω_x_, ω_y_, ω_z_). The data were sampled at a rate of 12,000 Hz, ensuring fine-grained temporal resolution. Joint controllers received commands via the ROS joint command topic, enabling dynamic articulation and interaction under realistic operating conditions. The collected data were stored in the CSV format for subsequent processing and analysis.

The data were extracted for six classes, designed to simulate three distinct paths of the robotic arm in a welding scenario. Each path consisted of a “good signal” and a “bad signal”, which were artificially labeled based on the damping values (dp) of the robotic arm. A high damping value of 1000 kg/s was used to simulate faulty or degraded system conditions, while a low damping value of 200 kg/s represented optimal operating conditions. As mentioned in the next part, these classification thresholds were fixed based on the work and the insights of previous research work.

Several studies have explored the role of damping in ensuring stable and efficient robotic operations, providing insights into selecting an optimal damping coefficient for different applications. Erickson et al. estimate damping coefficients in the range of 200 kg/s for stable force interactions in robotic systems, emphasizing the importance of appropriate damping selection to avoid excessive oscillations or sluggish performance [39]. Similarly, recent work on damping ratio prediction for Cartesian impedance-controlled robots suggests that optimal damping ratios typically range between 0.2 and 1.0, depending on the task and system constraints. These findings align with the approach of iteratively tuning damping coefficients to balance agility and stability, ensuring optimal system response without excessive energy dissipation [40,41]. Additionally, studies on damping matrix designs for robot manipulators highlight the necessity of fine-tuning damping values through modal analysis and experimental validation, reinforcing the idea that the selection of damping is application-dependent rather than universally fixed [42]. Given these findings, our approach was to incorporate these data-driven insights to manually mark the different sensor signals into “good signal” and “bad signal” classes.

To transform the raw sensor signals into image representations suitable for machine learning, a convolutional autoencoder-based feature extraction method was employed. The autoencoder comprised an encoder that reduced the high-dimensional sensor data into a latent representation and a decoder that reconstructed the input from this compressed representation. Mathematically, the encoder transformation is given by the following Equation (2):

(2) $$f_{encoder}(x)=\sigma (W_{e}x+b_{e}),$$

where x is the input sensor signal, $W_{e}$ and $b_{e}$ are the encoder weights and biases, respectively, and σ is the activation function. The decoder reconstructed the input as in the following Equation (3):

(3) $$\hat{x}=f_{decoder}(x)=\sigma (W_{d}z+b_{d}),$$

where $\hat{x}$ is the reconstructed output and z is the latent representation. The weights ($W_{e}$, $W_{d}$) and biases ($b_{e}$, $b_{d}$) are learned parameters that are updated during training via backpropagation and gradient descent while optimizing the reconstruction loss as explained via Equation (4). Here, $W_{e}$ and $b_{e}$ transform the input into the latent space, while $W_{d}$ and $b_{d}$ map the latent representation back to the original input space.

The autoencoder was trained to minimize the reconstruction loss, as in the following Equation (4):

(4) $$L_{reconstruction}={\left\|x-\hat{x}\right\|}^{2}$$

**Figure 2:**
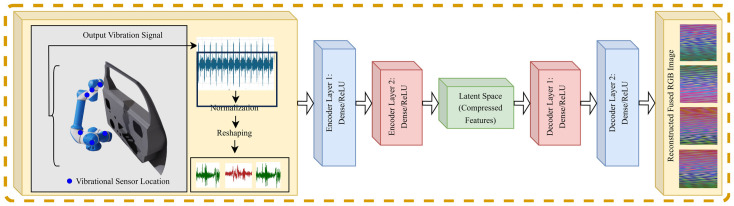
Convolutional autoencoder-based sensor signal to fused RGB image construction.

This ensured that the latent space z retained critical features. These latent features were then used to generate 2D images, preserving essential vibration and motion patterns for subsequent analysis. The workflow for this part is illustrated in Figure 2.

Next, we employ a vision transformer (ViT) to extract high-dimensional features from the RGB images. The vision transformer, leveraging self-attention mechanisms, captures both local and global relationships within the image, enabling robust feature representation as per Equations (5a) and (5b) followed by (6a) and (6b), and saves a 768-dimensional feature vector for each image.

(5a) $$x_{p}=\mathrm{reshape}\left(I,\left(N,P\times P\times 3\right)\right),$$

(5b) $$z_{0}=\left[x_{p}^{1}E;x_{p}^{2}E;\ldots ;x_{p}^{N}E\right]+P,$$

where P is the positional encoding matrix and N refers to the number of image patches.

$x_{p}$ is patch embeddings and E is the linear embedding matrix, and *p* is the positional encoding matrix.

(6a) $$z^{l+1}=\mathrm{MSA}\left(\mathrm{LN}\left(z^{l}\right)\right)+z^{l}$$

(6b) $$z^{l+1}=\mathrm{MLP}\left(\mathrm{LN}\left(z^{l+1}\right)\right)+z^{l+1}$$

where $z^{l}$ is the input to the layer at level l. MSA represents the multi-head self-attention mechanism; LN is layer normalization, l is the transformer layer index, and MLP represents the feed-forward network. More information on the importance of operational grouping is provided in the next section.

**Figure 3:**
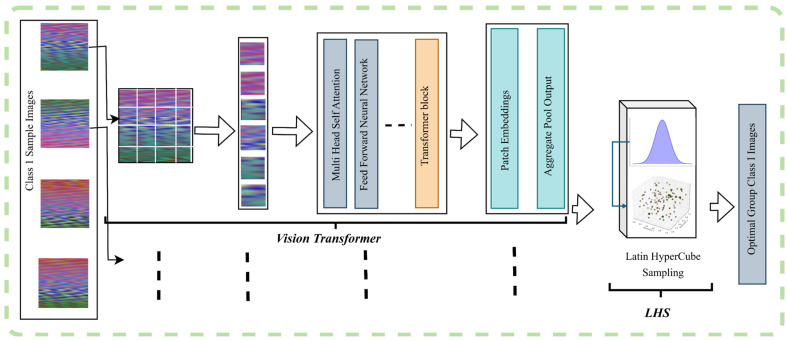
Operational grouping method with the vision transformer and LHS method.

Subsequently, we use Latin hypercube sampling (LHS) to group the images based on their feature distribution, ensuring that the selected subset exhibits maximum data variation. This sampling strategy allows us to identify a diverse and representative set of 5 or 10 images called an operational group (G(D_support_)), depending on the shot configuration in few-shot learning, ensuring that the model is exposed to the most informative examples, which during the next step is fed on to the meta-testing loop as a support set. Figure 3 provides an overview of the feature extraction process with the vision transformer and the deployment of LHS for operational grouping after that. Notably, as previously mentioned, few-shot approaches can explicitly normalize the imbalance of a dataset because of the very limited number of defective data samples (1~10 in most cases) they require for training, making the imbalance ratio close to 1 in most cases, as explained in the next two equations.

(7a) $$Overall\;Imbalance\;Ratio\left({IR}_{overall}\right)=\frac{N_{normal}}{N_{defective}}\approx 10\;\;where\;N_{normal}\gg \;N_{defective}\;,$$

(7b) $$Few\;Shot\;Imbalance\;Ratio\left({IR}_{few-shot}\right)=\frac{M_{normal}}{M_{defective}}\approx 1\;\;where\;M_{normal}\approx \;M_{defective},$$

### 3.2. Ensemble MAML Framework

Model-agnostic meta-learning (MAML) is a versatile meta-learning algorithm designed to train models that can rapidly adapt to new tasks using a minimal amount of task-specific data and only a few gradient updates. MAML achieves this by optimizing the initial parameters θ such that effective task-specific generalization can be achieved with minimal adaptation. Each task is divided into a support set for task-specific adaptation and a query set to evaluate the adapted parameters. This enables MAML-trained models to adapt quickly without requiring extensive retraining, making it particularly useful for certain applications, like fault classification and predictive maintenance.

MAML operates through two main optimization steps, namely the inner loop, where task-specific adaptation is performed using the support set, and the outer loop, where meta-parameters θ  are optimized across tasks based on their performance on the query set. During the inner loop, the parameters θ are updated for each task $T_{i}$ using gradient descent on the task-specific loss as in the following Equation (8):

(8) $$\theta_{i}^{\prime }=\theta -\alpha \nabla_{\theta }L_{T_{i}}^{\mathrm{support}}$$

where $\theta_{i}^{\prime }$ are task-specific parameters after adaptation and α represents the inner-loop learning rate. This learning rate is critical for controlling how quickly the model adapts to the task-specific data. However, α introduces significant sensitivity into the system, as excessively high or low values can result in overfitting or poor adaptation, respectively.

The outer loop optimizes the meta-parameters θ based on the performance of the adapted parameters $\theta_{i}^{\prime }$ on the query set as in the following Equation (9):

(9) $$L_{\mathrm{meta}}=\sum_{i}L_{T_{i}}^{\mathrm{query}}$$

where $L_{\mathrm{meta}}$ is the meta-learning loss, which we aim to optimize for fast adaptation to new tasks, and $L_{T_{i}}^{\mathrm{query}}$ represents the query set loss for the individual task $T_{i}$. The total meta-learning loss aggregates the query set losses across tasks. The optimization of the meta-learning loss is important because it optimizes the meta-parameters, making the model generalize better across different tasks and allowing it to adapt quickly to new unseen tasks with only a few examples.

The meta-parameters θ are updated using the gradient of the meta-loss as in the following Equation (10):

(10) $$\theta \leftarrow \theta -\beta \nabla_{\theta }L_{\mathrm{meta}}$$

where β is the outer-loop learning rate. Similar to α, β introduces additional sensitivity to the system, as improper tuning can destabilize the training process or slow convergence.

The inherent hypersensitivity of MAML to these learning rates, namely α and β, presents a significant challenge. A small deviation in α can cause inefficient adaptation in the inner loop, while an imbalanced β can lead to unstable or divergent updates in the outer loop. This instability becomes more pronounced as the number of epochs increases, often resulting in erratic learning curves and suboptimal task generalization.

To address the instability caused by the sensitivity of MAML to its learning rates (α and β) in the inner and outer loops, we introduce an ensemble layer. This ensemble combines predictions from multiple MAML models, each trained with distinct combinations of learning rates. By aggregating the outputs through a majority voting mechanism, the ensemble reduces the influence of hyperparameter sensitivity and significantly improves the robustness and generalization of the model. Each model within the ensemble learns independently, resulting in diverse decision boundaries that collectively mitigate the instability typically observed in meta-learning frameworks.

Let the ensemble consist of *n* = 6 models, where each model is denoted as $f_{i}(x;\;\alpha_{i},\;\beta_{i})$. Here, x represents the input data, $\alpha_{i}$ and $\beta_{i}\;$ are the inner-loop and outer-loop learning rates for the i-th model, respectively, and $f_{i}(x;\;\alpha_{i},\;\beta_{i})$ outputs the class prediction of the i-th model. By assigning each model a unique combination of learning rates, the ensemble ensures that a variety of learning dynamics are captured, leading to a more stable and effective prediction system.

The prediction from each model for a given input x is represented as in the following Equation (11):

(11) $$y_{i}=f_{i}(x;\alpha_{i},\;\beta_{i})$$

where $y_{i}$ belongs to the set of class labels {1, 2, …, M}, with M being the total number of classes. The final prediction from the ensemble, denoted as $y_{ensemble}$, is determined using a majority voting mechanism. This can be expressed mathematically as in the following Equation (12):

(12) $$y_{ensemble}=argmax_{j\in 1,2,\ldots ,M}\sum_{i=1}^{N}I\left(f_{i}\left(x;\alpha_{i},\beta_{i}\right)=j\right),$$

where $I(f_{i}(x;\;\alpha_{i},\;\beta_{i})=j)$ is an indicator function that equals 1 if the i-th model predicts class j, and 0 otherwise. This equation aggregates the votes for each class across all models and selects the class with the highest count as the final prediction.

**Figure 4:**
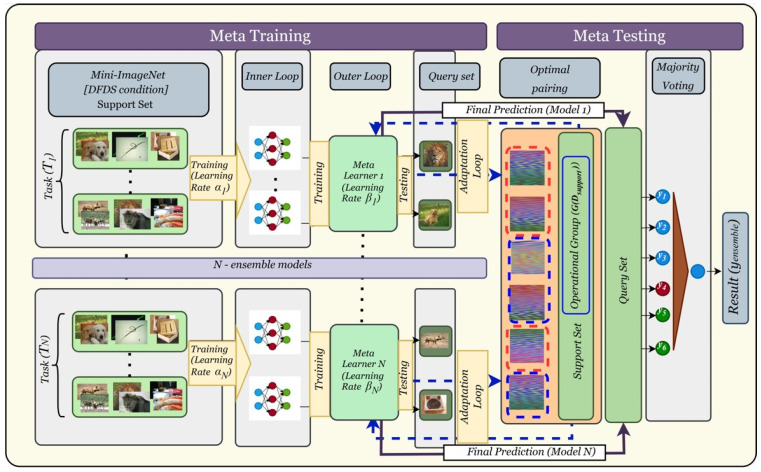
Ensemble MAML workflow.

The ensemble models are constructed with distinct learning rate configurations to capture diverse behaviors. For instance, the six models could be configured as $f_{1}(x;\;\alpha_{1},\;\beta_{1})$, $f_{2}(x;\;\alpha_{2},\;\beta_{2})$, and so on, up to $f_{6}(x;\;\alpha_{6},\;\beta_{6})$. This diversity in learning rates ensures that the ensemble spans a wide range of learning dynamics, effectively countering the hypersensitivity of MAML to specific hyperparameter settings. Each model operates independently, and their outputs are collectively analyzed in the ensemble layer. The workflow of this part is presented in Figure 4, showcasing the strategy for mitigating sensitivity to hyperparameters and improving the stability and accuracy of the model during training and adaptation.

This ensemble approach has several advantages. First, it reduces sensitivity by smoothing out the fluctuations caused by learning rate instability in individual models. Second, it improves generalization ability by aggregating predictions from models with diverse configurations, enhancing overall decision-making. Lastly, the robustness of the system is significantly improved, as the majority voting mechanism ensures that outliers or unstable predictions from individual models do not overly influence the final output. In essence, the ensemble mechanism effectively addresses the challenges posed by learning rate sensitivity while retaining the adaptability and efficiency of the MAML framework, making it particularly suitable for real-world applications in such tasks as fault prediction and classification in industrial robotics.

The algorithm for the proposed framework is as follows (Algorithm 1):
**Algorithm 1** Ensemble-Based MAML with Operational Grouping in Meta-Test Phase
**Require:** Dataset of tasks $\left\{T_{i}\right\}$, learning rates ${\left\{\alpha_{i},\beta_{i}\right\}}_{i=1}^{N}$ for N ensemble models, number of inner loop steps K, operational grouping strategy G.
**Ensure:** Final ensemble prediction $y_{\mathrm{ensemble}\;}$.1:**Meta-Train Phase:** Initialize N MAML models ${\left\{f_{i}\left(\theta_{i}\right)\right\}}_{i=1}^{N}$ with random parameters ${\left\{\theta_{i}\right\}}_{i=1}^{N}$.2:**for** each task $T_{i}$ from the meta-training dataset **do**3:  Split $T_{i}$ into support set $D_{\mathrm{support}\;}$ and query set $D_{\mathrm{query}\;}$.4:  **for** each model $f_{i}\left(\theta_{i}\right)$ in the ensemble **do**5:    Compute loss $L_{\mathrm{support}\;}=\frac{1}{\left|D_{\mathrm{nupport}\;}\right|}\sum_{(x,y)\in D_{\mathrm{support}\;}}l\left(f_{i}\left(x;\theta_{i}\right),y\right)$.6:    Adapt $\theta_{i}$ via K gradient steps: $\theta_{i}^{\prime }=\theta_{i}-\alpha_{i}\nabla_{\theta_{i}}L_{\mathrm{support}\;}$.7:    Evaluate loss $L_{\mathrm{query}\;}=\frac{1}{\left|D_{\mathrm{query}\;}\right|}\sum_{(x,y)\in D_{\mathrm{query}\;}}l\left(f_{i}\left(x;\theta_{i}^{\prime }\right),y\right)$.8:    Update meta-parameters: $\theta_{i}\leftarrow \theta_{i}-\beta_{i}\nabla_{\theta_{i}}L_{\mathrm{query}\;}$.9:**  end for**10:**end for**11:**Meta-Test Phase:**12:**for** each task $T_{j}$ from the meta-test dataset **do**13:  Split $T_{j}$ into support set $D_{\mathrm{support}\;}$ and query set $D_{\mathrm{query}\;}$.14:  Apply Operational grouping $G:D_{\mathrm{support}\;}^{G}=G\left(D_{\mathrm{support}\;}\right)$.15:  **for** each model $f_{i}\left(\theta_{i}\right)$ in the ensemble **do**16:    Compute loss $L_{\mathrm{support}\;}^{G}=\frac{1}{\left|D_{\mathrm{nupport}\;}^{G}\right|}\sum_{(x,y)\in D_{\mathrm{kupport}\;}^{G}}l\left(f_{i}\left(x;\theta_{i}\right),y\right)$.17:    Adapt $\theta_{i}:\theta_{i}^{\prime }=\theta_{i}-\alpha_{i}\nabla_{\theta_{i}}L_{\mathrm{support}\;}^{G}$.18:
  **end for**
19:  Aggregate predictions via majority voting: $y_{\mathrm{ensemble}\;}$$arg\underset{j\in \{1,\ldots ,M\}}{max}\sum_{i=1}^{N}I\left(f_{i}\left(x;\theta_{i}^{\prime }\right)=j\right)$.20:**end for**21:**Return** $y_{\mathrm{ensemble}\;}$.

## 4. Experimental Setup

The experiments were conducted on a system equipped with an Intel(R) Xeon(R) w7-3445 processor running at 2.59 GHz and 64 GB of RAM, with 16 GB allocated for an NVIDIA A4000 GPU (HP, Palo Alto, CA, USA). This hardware setup enabled efficient parallel processing for running multiple MAML models.

Six separate models were trained and evaluated, each using a unique combination of inner-loop (α) and outer-loop (β) learning rates. The specific values for these parameters obtained through LHS inside the bounds α ∈ (0.001, 0.003) and β ∈ (0.02, 0.05) are detailed in Table 1. During the meta-training phase, data from the MiniImageNet dataset were used for both the support and query sets. In the meta-testing phase, the operationally grouped support data were employed to ensure maximum information intake, while the query set was used for evaluation. In our custom dataset, there were six classes depending on the three path scenarios, as mentioned previously.

The experimental setup kept several key hyperparameters constant to ensure a fair comparison across different configurations. The meta-learning rate (meta_lr) was set to 0.003, providing a stable yet effective learning pace for updating the meta-learner across tasks. The fast learning rate (fast_lr) was set to 0.5, enabling rapid adaptation within inner-loop updates during task-specific learning. A meta-batch size of 32 was used, ensuring sufficient tasks per iteration to enhance generalization across different scenarios. The model underwent 10 adaptation steps, allowing it to refine its representations progressively within each task. Finally, the training was conducted over 600 iterations, ensuring adequate exposure to diverse tasks while maintaining computational efficiency.

Three experimental scenarios were tested: six-way one-shot, six-way five-shot, and six-way ten-shot, allowing us to evaluate the model’s performance under varying levels of data availability. Details of the datasets used for meta-training and meta-testing are summarized in Table 2.

**Figure 5:**
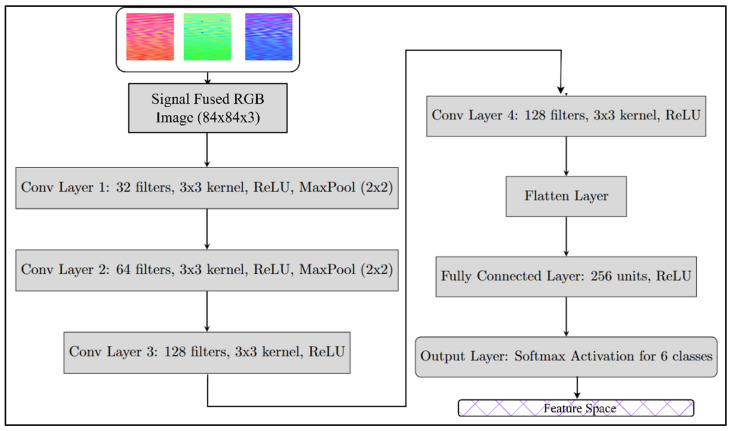
The CNN network used inside the EMOG for classification.

The CNN network structure used for 6-class image classification in MAML is illustrated in Figure 5. This network serves as the feature extractor and classifier for all six models.

## 5. Results and Discussion

**Figure 6:**
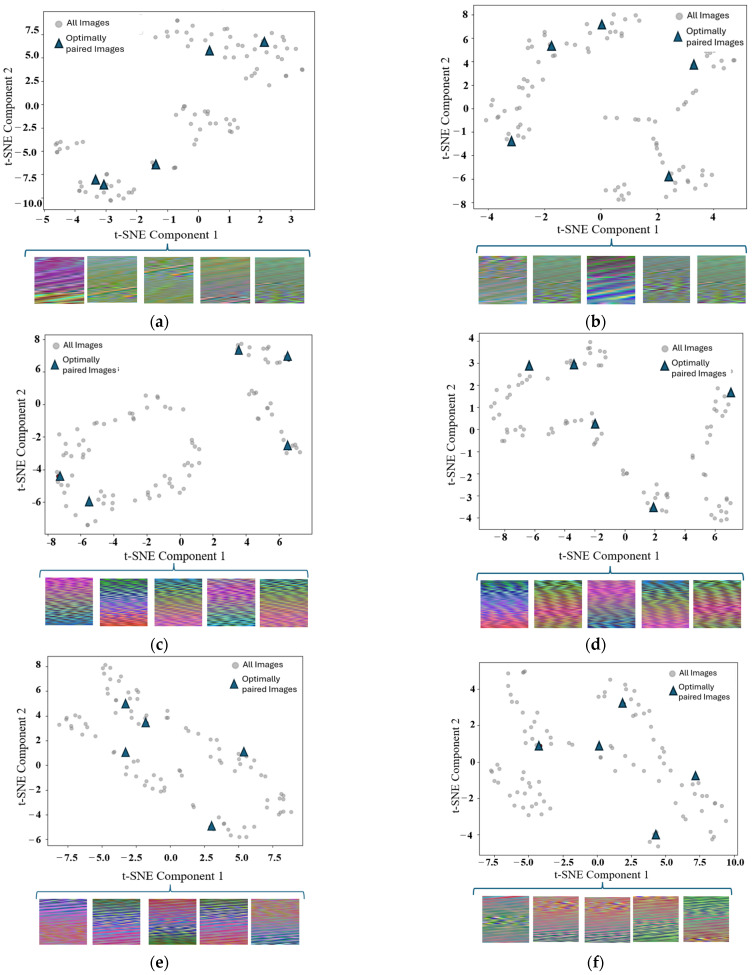
Operationally grouped images for the 5-shot case. (**a**) Class—path1, dp = 250; (**b**) class—path1, dp = 1000; (**c**) class—path2, dp = 250, (**d**) class—path2, dp=1000; (**e**) class—path3, dp = 250; (**f**) class—path3, dp = 1000.

In the analysis presented in Figure 6, the operational grouping strategy for the t-SNE visualization effectively showcases the distribution of selected images within the feature space. For the five-shot case, as depicted in the visualization, the points representing the selected images are well spread across the feature space. This indicates that the grouping captures a wide range of information from the dataset, ensuring maximum diversity in the support set and enabling the model to better generalize during the adaptation process.

Similarly, for the 10-shot case, the spread of points is consistent with the 5-shot observations but with even more excellent coverage of the feature space. This reinforces the notion that higher shot counts allow for more comprehensive information intake. Leveraging this grouping strategy enriches the model’s support sets with varied and representative examples, ensuring improved learning outcomes during evaluation.

The results of the experiments, as provided in Table 3, consistently highlight the superior performance of EMOG across all evaluation metrics, especially in multi-shot scenarios where the benefits of operational grouping are more pronounced. Regarding testing accuracy, EMOG demonstrates a significant advantage, achieving 71.4% in the one-shot case, which is approximately 7% higher than MAML’s 64.4% and 15% higher than ANIL’s 56.3%. This margin becomes even more pronounced in the five-shot case, where EMOG reaches 85.2%, outperforming MAML by over 11%. The trend continues in the 10-shot case, where EMOG achieves an impressive 93.8%, maintaining a substantial 11% lead over MAML’s 82.8%. These results indicate that EMOG effectively leverages the increased support set size, improving in terms of accuracy as the number of shots increases.

In terms of precision, EMOG consistently exhibits stronger performance across all settings. For the five-shot case, EMOG achieves a precision of 84.6%, which is 11.4% higher than MAML’s 73.2%. In the 10-shot setting, EMOG reaches 93.1%, surpassing MAML by 13.7%. This suggests that EMOG is not only making accurate predictions but is also confident in its predictions, benefiting from the diversity introduced through operational grouping. The trend is similar for recall, where EMOG consistently outperforms other methods. In the one-shot case, EMOG achieves a recall of 70.4%, outperforming MAML’s 62.7% by 7.7%. This improvement is even more noticeable in the five-shot case, with EMOG reaching 84.3%, 11.5% higher than MAML’s 72.8%. For the 10-shot case, EMOG achieves a recall of 92.9%, significantly surpassing MAML by 13.1%.

F1 score, which balances precision and recall, highlights EMOG’s overall robustness. In the one-shot case, EMOG achieves an F1 score of 70.6%, approximately 6.7% higher than MAML. In the five-shot case, EMOG records an F1 score of 84.4%, significantly improving over MAML’s 73.0%. In the 10-shot case, EMOG achieves 93.0%, maintaining a 13.4% lead over MAML. These results show that EMOG performs well in accuracy and effectively balances its predictions.

**Figure 7:**
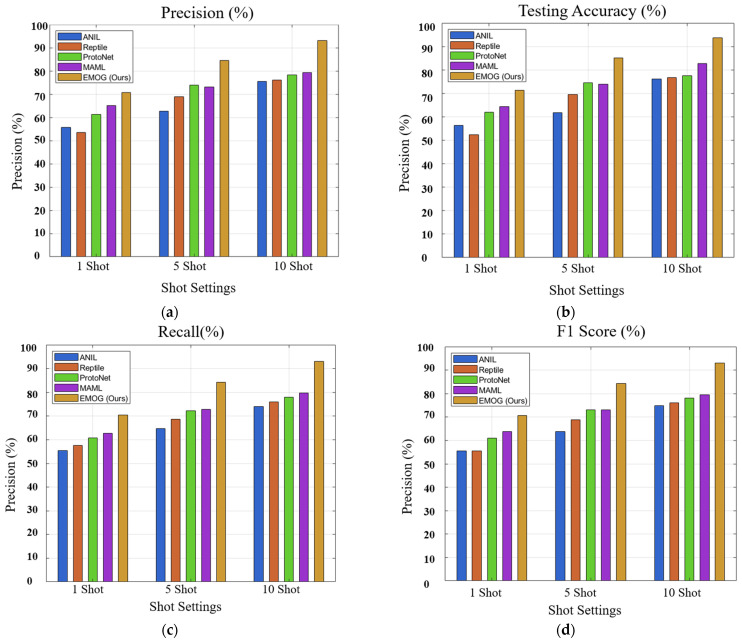
Comparative analysis of different few-shot learning methods. (**a**) Precision graph. (**b**) Accuracy graph. (**c**) Recall graph. (**d**) F1 score graph.

The results from the comparative study provided in Figure 7 suggest that EMOG’s operational grouping strategy is particularly beneficial in the 5-shot and 10-shot cases, where there is more opportunity to exploit the diversity and structure of the support set. In comparison, the one-shot case shows more modest improvements, indicating that while EMOG is effective even with limited data, its potential is fully realized when larger support sets are available.

**Figure 8:**
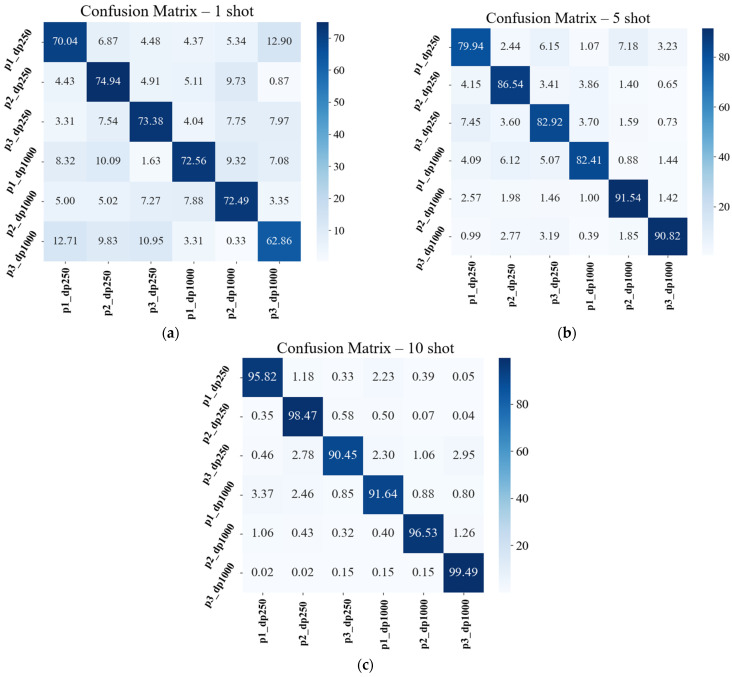
Confusion matrices of the EMOG method: (**a**) 1-shot case; (**b**) 5-shot case; (**c**) 10-shot case.

The confusion matrices, provided in Figure 8, for the EMOG framework demonstrate strong classification performance across different shot settings (1-shot, 5-shot, and 10-shot), with diagonal values consistently indicating accuracies above 70% for most classes. As the number of shots increases, the classification accuracy improves significantly, with 10-shot learning achieving over 95% accuracy for nearly all classes. This trend highlights the effectiveness of increasing the support set samples in reducing class confusion and improving model generalization.

However, certain classes, such as p4_dp1000, exhibit higher misclassification rates, particularly in the lower-shot settings. This suggests potential feature overlap between specific classes, leading to misclassification. The confusion is more pronounced in the one-shot and five-shot settings, where limited training samples may result in insufficient feature separation. As seen in the one-shot matrix, misclassification values for non-diagonal elements remain relatively high, indicating difficulty distinguishing between some classes due to shared feature similarities.

With 5-shot and 10-shot settings, the classification performance improves significantly, as evidenced by reduced off-diagonal misclassifications and sharpened class boundaries, thus reconfirming the effectiveness of the operational grouping strategy.

## 6. Conclusions

The EMOG method significantly improves few-shot learning performance across multiple metrics, with notable gains in 5-shot and 10-shot scenarios. Its operational grouping strategy enhances adaptation and feature separation, making it more effective for predictive maintenance (PdM) than traditional meta-learning approaches. A key advantage of EMOG is its ability to address the two major shortcomings of MAML in PdM. It stabilizes inner- and outer-loop learning rates, improves convergence in lower epochs, and increases information intake from highly imbalanced datasets. By leveraging operational grouping, EMOG ensures better utilization of minority class representations, leading to more balanced learning. The confusion matrices further validate EMOG’s effectiveness, showing reduced misclassification rates and stronger class separability as the support set grows. Performance gains over MAML, ProtoNet, ANIL, and Reptile highlight EMOG’s superior ability to generalize from limited data, making it a robust, scalable, and efficient solution for few-shot learning in predictive maintenance.

## Figures and Tables

**Table 1:** Different MAML model configurations in EMOG.

Model No.	Inner-Loop Learning Rate (α)	Outer-Loop Learning Rate (β)
M1	0.002264	0.040161
M2	0.001987	0.046838
M3	0.001475	0.001475
M4	0.001008	0.036277
M5	0.002848	0.029295
M6	0.002592	0.024300

**Table 2:** Dataset details used for meta-testing.

Sample Number	Path 1 (A-B)	Path 2 (B-C)	Path 3 (C-D)
Correct damping (dp = 250)	50	50	50
Defective damping (dp = 1000)	50	50	50

**Table 3:** Comparative results of different few-shot scenarios.

	Testing Accuracy (%)			Precision (%)			Recall (%)			F1 Score (%)		
Shot number	1	5	10	1	5	10	1	5	10	1	5	10
ANIL	56.3	61.8	76.2	55.8	62.8	75.5	55.4	64.8	74.1	55.6	63.8	74.8
Reptile	52.4	69.6	76.8	53.5	69	76.2	57.6	68.6	76	55.5	68.8	76.1
ProtoNet	61.9	74.6	77.6	61.3	73.9	78.4	60.8	72.2	77.9	61.0	73.0	78.1
MAML	64.4	73.9	82.8	65.1	73.2	79.4	62.7	72.8	79.8	63.9	73.0	79.6
EMOG (Ours)	71.4	85.2	93.8	70.8	84.6	93.1	70.4	84.3	84.3	70.6	84.4	93.0

## Data Availability

The data can be accessed upon request to the corresponding author.
